# Attitudes toward brushing children's teeth—A study among parents with immigrant status in Norway

**DOI:** 10.1111/ipd.12683

**Published:** 2020-07-11

**Authors:** Manal Mustafa, Elwalid F. Nasir, Anne Nordrehaug Åstrøm

**Affiliations:** ^1^ Oral Health Centre of Expertise in Western Norway Hordaland, Bergen Norway; ^2^ King Faisal University SA University of Science and Technology Omdurman Sudan; ^3^ Department of Clinical Dentistry Faculty of Medicine University of Bergen Bergen Norway

**Keywords:** attitude, children, immigrant, knowledge, toothbrushing

## Abstract

**Background:**

Early childhood caries (ECC) is a common chronic childhood disease with multifactorial etiology including poor parental dietary and hygiene behaviors.

**Aim:**

This study aimed to assess toothbrushing‐related perceptions among parents with immigrant background living in Norway.

**Design:**

A structured interview was performed with immigrant parents to assess their oral health‐related knowledge, beliefs, and attitude toward toothbrushing. Immigrant parents of non‐Western origin with newborn infants (0‐6 months) were included in this study.

**Results:**

Of those interviewed, 66% chose to participate and they were found to have an average favorable attitudes, subjective norms, and strong perceptions of control related to child's tooth brushing with reported means of (3.3), (3.6), and (4.6), respectively. They had on average low indulgence (mean 7.8) with respect to this behavior and a relatively high level of knowledge (mean 6.9). Parents with strong intention toward toothbrushing (61%) had on average more frequent oral hygiene behavior than parents with weak intentions.

**Conclusion:**

Parents with non‐Western origin have adequate knowledge and intention toward toothbrushing, although some have an unsatisfactory attitude, which might affect the oral health of their children negatively. Culture and habits are contributing factors in ECC and should be addressed in oral health prevention policies.

**Why this paper is important for pediatric dentists**

- Early childhood caries (ECC) is a multifactorial disease, and this work addresses the culture and habits as contributing factors and awareness of these factors might help pediatric dentists adopting educational prevention policies.
- Among others, knowledge of perceptions toward children's regular toothbrushing might help in oral health prevention procedures implemented by pediatric dentists.
- Pediatric dentists can reduce the burden of this disease, by understanding parents' perceptions, breaking the communication barrier, and consequently diminish existing oral health inequalities.

## 1 INTRODUCTION

As of March 9, 2020, people with immigrant status constituted a substantial part (about 18.2%) of the Norwegian population (Statistics Norway (SSB), https://www.ssb.no).^1^ Although experiencing a vulnerable transitional period in their lives, most immigrants represent an increasing health and societal challenge to their host society.^2^, ^3^ Several studies have shown that people with immigrant background have more socioeconomic difficulties, poorer well‐being status, and suffer more from lifestyle‐ and diet‐related disorders compared to the native population.^4^ Moreover, evidence suggests that children of immigrant parents are at greater risk of diverse health problems than are their host country counterparts.^4^

According to the American Academy of Pediatric Dentistry, early childhood caries (ECC) is any form of caries (cavitated and non‐cavitated) occurring in the primary dentition of children aged 6 years or less.^5^ Non‐European‐born parents and parents who had immigrated in or after their 20s were 2 to 4 times more likely to have a child with ECC than European parents or parents who immigrated at a younger age.^6^ A Norwegian study revealed that children of immigrant parents aged 3‐5 years have more caries than native Norwegian children.^7^ Studies from other Scandinavian countries^4^, ^8^, ^9^ and from the United States^10^ have reported similar findings. Ethnic inequalities in children's and adolescents' oral health does not seem to be fully explained by socioeconomic differences.^4^

According to multilevel conceptual models, the prevalence of ECC varies according to risk factors at the community, family, and individual level, such as culture, ethnicity, socioeconomic status, dietary and oral hygiene practices, as well as parental knowledge, values, and attitudes.^11^, ^12^ A general trend worldwide suggests that families of low socioeconomic and educational status are predisposed to experience ECC.^13^ As a multifactorial disease, ECC, however, also involves individual level factors such as psychological conditions, that is, parental depression and anxiety, as well as lifestyle. The association between poor oral hygiene practices and ECC, for instance in terms of delayed introduction of tooth brushing, has also been documented.^14^ It is acknowledged that parental dental health behavior influences that of their children and associations between mother's and toddler's tooth brushing behavior has been documented in a previous study.^15^ Poor knowledge and unfavorable attitudes toward dental health are associated with increased risk of ECC^16^ and are more frequently reported across time among parents with than without immigrant background.^17^ A recent Norwegian study reported that parental attitudes were more strongly related to children's caries status than were children's own oral hygiene behavior.^17^

According to socio‐cognitive theory, attitudes and perceptions influence caretakers' decision to care for their children's teeth and their actual caring behavior.^18^ The theory of planned behavior (TPB) posits that people are quite rational making systematic use of information available to them. According to the TPB, intention to perform an oral health‐related behavior, such as tooth brushing, is the immediate precursor of actual tooth brushing behavior. Intention to brush is, in turn predicted by attitudes, subjective norms and perceived behavioral control toward toothbrushing. The TPB has successfully accounted for a high proportion of variance in several health‐related behaviors including those related to oral health.^19^, ^20^

A recent systematic review of caries preventive strategies in immigrant children revealed that an early child/mother approach focusing on broader oral health education of mothers might be effective.^21^ Evidence, however, suggests that it is difficult to influence oral health‐related attitudes in a favorable direction among immigrants.^21^ This may be due to culturally inappropriate service programs and one might argue that traditional health education in the field of dentistry has failed to account for the entire range of relevant perceptions and attitudinal factors in target populations. Thus, information about behavioral mediators, such as family values, parental attitudes, and perceptions, provides a valuable tool in the planning, implementation, and evaluation of culturally appropriate caries preventive programs. Such information is rare when it comes to immigrant parents of young children in Norway.

Focusing on immigrant parents of children aged 0‐6 months attending child public health centers for child vaccination, the purpose of this study was to examine.

- To what extent immigrant parents' oral health‐related knowledge, attitudes toward a child's toothbrushing, and their own oral hygiene behavior vary with sociodemographic characteristics
- To what extent parents' intention to brush a child's teeth is predicted by Ajzen's model in terms of attitudes, subjective norms and perceived behavioral control in addition to parental oral health‐related knowledge, indulgence, and their own oral hygiene behavior.

## 2 METHODS

This study constitutes the baseline survey of a prospective caries preventive intervention program conducted among children aged 0‐6 months and their accompanying caregivers with immigrant background who attended public health child service centers in Bergen for childhood vaccination.

The study received ethical approval from the Regional Committee for Medical Research Ethics (2015/2037/REK vest).

Five of 8 geographically distributed health centers in Bergen were selected to participate in the baseline survey. The needed sample size was estimated to be 200 parent/child pairs by using Satterthwaite's *t* test assuming unequal variance between individuals having satisfactory compared to unsatisfactory knowledge. The mean of knowledge, attitude toward hygiene, diet, and parental indulgence together were used for sample size calculation. The total sample of this study consisted of 233 immigrant parents and their 0‐6 month's old children who attended the selected health centers, between October 2017 and December 2018, for vaccination. Immigrant parents with non‐Western origin, either mother, father, or both born outside Norway, were included in this study. Non‐Western (NW) background implies origin from NW countries including Eastern Europe, Asia, Africa, Turkey, and South and Central America. All immigrant parents attending the healthcare centers during the study period and who satisfied the inclusion criteria were invited to participate. They were included if they were willing to join and if they were able to understand and consent. An interpreter was used for those with limited Norwegian‐English language skills and for those who did not have a family member that could interpret the questions at the time of data collection. An experienced dental professional researcher explained the purpose of the study and provided participating immigrant parents with a face‐to‐face interview whereas waiting the recommended time after vaccination before discharge. The interview was conducted in Norwegian. Participation was voluntary, and written consent was obtained from all participants. Completion of the interview took between 15 and 20 minutes.

### 2.1 The study interview

The main questions incorporated each component of Ajzen's theory of planned behavior in terms of attitudes, subjective norms, perceived behavioral control, and intention related to brushing children's teeth twice a day in the future.^18^ Ajzen stressed the importance that all behavioral predictors are self‐referent and measured at the same specificity.^18^ All questions incorporated in the interview were developed in accordance with these recommendations. The study questions were adapted from an existing validated instrument developed to assess the oral health knowledge and attitudes of parents in Norway where immigrants were part of the study participants.^17^ Some few questions were revised to fit this study population. An expert panel consisting of clinicians, academics, reviewed content validity, and their agreement on the survey items were sought through qualitative feedback. Minor revisions of items were undertaken based on the feedback received. The questions were further refined after a pilot study in 20 NW immigrants caretakers not included in the main study sample. Interviews with a separate focus group (healthcare workers) at the healthcare centers were conducted to ensure quality relevance and adjustment to local circumstances. Structured interviews (in Norwegian/ English/ other different languages with use of an interpreter) were performed with immigrant parents to assess family oral hygiene, dietary behaviors, oral health‐related knowledge, and beliefs.

### 2.2 Measures

Basic demographic information consisting of parental age, country of origin and period of stay in Norway, mother/fathers' level of education, and employment status was also collected.

### 2.3 Oral health‐related knowledge

An additive sum score was constructed from 10 single items with responses given as (1) true and (0) false/unknown. The sum score ranged from four (low level of knowledge) to nine (high level of knowledge).

### 2.4 Components of TPB

*Intention to brush child's teeth twice a day* was measured by one item; ‘I intend to brush my child's teeth twice a day in the future’. Responses were given on a 5 point Likert's scale ranging from 1 (strongly agree) to 5 (strongly disagree). Since this variable was skewed, intention was dichotomized into (1) strong intention (including the original category 1) and (2) weak intention (including the original categories 2‐5).

*Attitudes toward brushing child's teeth twice a day were measured by 2 items:* ‘As a mother/father, I feel it is important to help brushing my child's teeth twice a day’ and ‘If I brush my child's teeth twice a day with fluoride toothpaste, my child will not get caries in future’. Each item was measured on a 5 point Likert scale ranging from (1) strongly agree to (5) strongly disagree. A sum additive score was constructed. The lower the score on this additive scale, the more favorable the attitude toward child's toothbrushing.

*Subjective norms toward child's toothbrushing* were measured by 2 items: ‘The rest of my family thinks it is important that I brush my child's teeth twice a day’ and ‘The people I know think it is important that I brush my child's teeth twice a day’. Each item was measured on a scale ranging from (1) strongly agree to (5) strongly disagree. A sum score was constructed by adding the two items. The lower the score on this additive scale, the more favorable the subjective norms toward child's toothbrushing.

*Perceived behavioral control* was measured by 3 items: ‘I feel I am capable of brushing my child's teeth twice a day in future’, ‘I cannot manage to brush my child's teeth twice a day in future’, and ‘I have no time to brush my child's teeth twice a day in future’. Each item was measured on a scale ranging from (1) strongly agree to (5) strongly disagree. After reversing the scores of the two latter items, an additive score was constructed. The lower the score on this additive scale, the stronger the perceived control toward child's toothbrushing.

### 2.5 Indulgence

Based on the health belief model,^22^ indulgence was measured by 2 items in terms of ‘If the child does not want to brush twice a day, I do not feel I should force him/her’ and ‘It is not worth it to battle with the child to brush his/ her teeth twice a day’. An additive score was constructed by adding the 2 items. The lower the score, the stronger the tendency of parental indulgence.

### 2.6 Parental oral health behaviors

The parents' own oral health‐related behavior was measured by 4 items: ‘How often do you brush your teeth’, ‘How often do you use the interdental cleaning device’, ‘How often do you use mouthwash’, and ‘How often have you been to the dentist within the last 5 years’.

The above mentioned questions were measured on a 5 point scale (every day, once‐twice during the week, once‐twice during the month, less than once a month, and never). ‘How often have you been to the dentist within the last 5 years’ was measured on a 5‐point Likert's scale (many times a year, twice a year, once a year, rarely than once a year, and did not visit the dentist within the last 5 years). An additive index was constructed by adding the items. The lower the score on this sum score, the more frequent the parental performance of oral hygiene behavior. The sum score was dichotomized based on a median split into (1) frequent oral hygiene performance and (2) less frequent oral hygiene performance.

### 2.7 Data analysis

The data were analyzed using SPSS version 24 (SPSS Inc). Descriptive statistics such as mean and standard deviation for continuous variables, frequency, and percentage for categorical variables were calculated and tabulated. Univariate analysis was performed using cross tabulation and chi‐square statistics. Independent sample *t* test was used to assess how sum scores of knowledge, attitudes, and own oral hygiene behavior varied according to parents' age, educational level, and length of stay in Norway. Multivariate analyses were performed using GLM (General linear models) with intention to brush as a fixed factor to study how attitudes, subjective norms, perceived control, knowledge, and oral hygiene behavior discriminated between parents with weak and strong intention to brush their child's teeth. Associations were quantified using eta coefficients. A two‐sided significance level of 5% was implied for all analyses.

## 3 RESULTS

Of the 351 invited to participate in the study, only 233 caregivers were willing to contribute and completed the interview, for a 66.4% response rate. The Table S1 described the participants' country of origin, and it was observed that more than 35% of the participating caretakers (both mothers and fathers) were born in Asia, 26% in Africa. Of the mothers included in the study, 52% (n = 123) were aged 30 years or less. The mean period of stay in Norway was 8.9 and 15.2 years among mothers and fathers, respectively, with 42% of mothers having lived more than 6 years in Norway. The number of children per family ranged between 1 and 4 children, and 36.5% of the study population reported having two children. More than half 55% of mothers reported higher education, and 58% were employed at the time of the interview (Table 1).

**Table 1:** Sample profile of immigrant caretakers (n = 466)

	Mother % (n)	Father % (n)
Years living in Norway		
6 y or less	57.9 (135)	31.3 (73)
More than 6 y	42.1 (98)	68.7 (160)
Employment		
Yes	58.2 (135)	82.8 (168)
No	41.8 (97)	17.2 (35)
Education level		
Lower	45.1 (105)	43.3 (101)
Higher	54.9 (128)	56.7 (132)
Age (Mean 30.35, SD 5.26)		
30 y or less	52.8 (123)	27.9 (65)
More than 30 y	47.2 (110)	72.1 (168)

As shown in Table 2, about 40% of parents recognized caries as a common disease among children and almost all study participants (96%) confirmed that frequent exposure to sugar‐containing food or drinks can lead to tooth decay. Ambiguity was reported about the correct time for intake of sugar‐containing food as about 74% of the parents disagreed both that intake of sugared foods should be given to children at meals and between meals. Moreover, about 80% mentioned that toothbrushing should start when the first tooth appears and they perceived that toothbrushing prevents caries. In contrast, nearly 75% of the study population believed that frequent baby breastfeeding at night could not lead to tooth decay (Table 2). Notably, most of the study participants were aware of the beneficial effect of fluoride toothpaste use.

**Table 2:** Frequency distribution of caretakers' oral health‐related knowledge % (n)

	% (n)
Caries common in small children	
Correct	39.5 (92)
False/I do not know	60.5 (141)
Frequent oral sugar causes caries	
Correct	96.1 (224)
False/I do not know	3.9 (9)
Sugared drinks do not cause caries	
Correct	7.3 (17)
False/I do not know	92.7 (216)
Nocturnal breastfeeding causes caries	
Correct	25.4 (59)
False/I do not know	74.6 (173)
Sugared food should be provided at meals	
Correct	25.9 (60)
False/I do not know	74.1 (172)
Sugared food should be provided between meal	
Correct	26.3 (61)
False/I do not know	73.7 (171)
Toothbrush when first tooth appear	
Correct	80.3 (184)
False/I do not know	19.7 (45)
Toothbrush when all teeth appear	
Correct	15 (35)
False/I do not know	85 (198)
Toothbrushing prevents caries	
Correct	84.5 (196)
False/I do not know	19.7 (45)
Fluoride causes caries	
Correct	7.3 (17)
False/I do not know	92.7 (216)

As shown in Table 3, immigrant parents had on average favorable attitudes (mean 3.3), subjective norms (mean 3.6), and strong perceptions of behavioral control (mean 4.6) related to child's tooth brushing. They had low average indulgence (mean 7.8) with respect to this behavior and a relatively high level of knowledge (mean 6.9). Sixty‐one percent of the participants confirmed strong intentions to brush their child's teeth.

**Table 3:** Mean scores, standard deviation (SD), and range with respect to sum scores of attitude, subjective norm, perceived behavioral control, indulgence, knowledge, and parents' own oral hygiene behavior

	Range high‐low	Mean (SD)
Maternal oral hygiene behavior	4‐16	9.0 (2.9)
Attitude toward brushing child's teeth 2 times a day	2‐6	3.3 (1.2)
Subjective norms toward brushing child's teeth twice a day	2‐10	3.6 (1.6)
Perceived control toward brushing child's teeth twice a day	3‐9	4.6 (1.7)
Indulgence regarding brushing child's teeth twice a day	2‐10	7.8 (1.7)
Oral health‐related knowledge	4‐9 (low‐high)	6.9 (1.1)
Strong intention to brush % (n)		60.9 (142)
Weak intention to brush % (n)		39.1 (91)

Note

Frequency (n) of participants with strong and weak intentions regarding child's toothbrushing.

Table 4 depicts the sociodemographic variation of scores regarding parental oral hygiene behavior, knowledge, and the TPB components. Parental oral hygiene behavior was on average more frequent in older than younger participants (mean value 8.3 vs 9.6, *P* < .001), as well as in participants having higher versus lower education (mean value 8.6 vs 9.4, *P* < .05). Attitudes and behavioral control perceptions were most favorable among participants who had lived in Norway for more than 6 years. Indulgence was on average stronger in parents with lower than higher education (mean value 7.6 vs 8.1, *P* < .05). Percentages of parents with strong intention toward child's toothbrushing were highest in mothers above the age of 30, those having lived more than 6 years in Norway and those with higher education. Those differences, however, were not statistically significant. Results from unadjusted independent sample *t* test revealed statistically significant (*P* < .05) associations between intention and the parental oral hygiene behavior, attitudes, subjective norms, perceived control, and indulgence (Table 5).

**Table 4:** Mean scores (SD) with respect to attitudes, subjective norms, perceived control, indulgence, knowledge, and oral hygiene behaviors by maternal age, length of residence in Norway, and education. (Independent sample *t* test)

	≤30‐y	≥30 y	≤6 y	>6 y	Low education	High education
	Mean (SD)	Mean ( SD)	Mean (SD)	Mean (SD)	Mean (SD)	Mean (SD)
Maternal oral hygiene behavior	9.6 (2.9)	8.3 (2.6)**	9.2 (3.0)	8.7 (2.6)	9.4 (3.0)	8.6 (2.7)*
Attitude toward brushing	3.3 (1.1)	3.2 (1.2)	3.5 (1.2)	3.1 (1.1)*	3.4 (1.2)	3.2 (1.2)
Subjective norms toward brushing	4.7 (1.6)	4.4 (1.6)	3.7 (1.6)	2.5 (1.8)	3.8 (1.8)	3.4 (1.4)
Perceived control toward brushing	3.7 (1.5)	3.5 (1.5)	4.8 (1.6)	4.4 (1.7)*	4.9 (1.6)	4.4 (1.6)*
Indulgence regarding brushing	7.7 (1.8)	8.0 (1.5)	7.8 (1.6)	8.1 (1.8)	7.6 (1.8)	8.1 (1.6)*
Oral health‐related knowledge	6.9 (1.0)	7.1 (1.3)	6.8 (1.1)	7.1 (1.6)	6.8 (1.1)	7.1 (1.1)

	%(n)	% (n)	% (n)	% (n)	% (n)	% (n)
Strong intention	56.9 (70)	65.5 (72)	56.3 (76)	67.3 (66)	54.3 (57)	66.4 (85)
Weak intention	43.1 (53)	34.5 (38)	43.7 (59)	32.7 (32)	45.7 (48)	33.6 (43)

* *P* < .05

** *P* < .001.

**Table 5:** Caretakers' attitudes to care for their child's toothbrushing, knowledge, and their own hygiene behavior by intention to brush child's teeth. Independent sample *t* test

	Strong intention	Weak intention
	Mean (SD)	Mean (SD)
Own oral hygiene behavior	8.6 (2.7)	9.4 (3.1)*
Attitudes toward child's toothbrushing	2.6 (1.0)	4.2 (0.9)**
Subjective norm regarding child's toothbrushing	3.1 (1.6)	4.4 (1.7)**
Perceived control regarding child's toothbrushing	3.8 (1.4)	5.8 (1.1)**
Indulgence regarding child's tooth brushing	8.1 (1.9)	7.4 (1.2)*
Knowledge oral health	7.1 (1.2)	6.9 (1.1)

* *P* < .05.

** *P* < .001.

To adjust for the associations between TPB constructs, knowledge, indulgence, and parents' own oral hygiene behavior, a multivariate GLM analyses were conducted with intention to brush the child's teeth as a fixed factor while controlling for sociodemographics. As shown in Table 6, the GLM analysis revealed statistically significant multivariable main effect of intention Wilks' Lambda *F* = 41.989, *P* < .001, partial *eta* 0.543. Estimated marginal means revealed that the components of TPB and indulgence discriminated statistically significantly (*P* < .05) and in the expected direction between parents with strong and weak intentions to brush their child's teeth. The mean values provide information about how frequently oral hygiene behaviors were performed and how favorably the various cognitive factors were held. Intenders had on average more frequent oral hygiene behavior, more favorable attitudes, subjective norms, and perceived control than parents with weak intentions. In contrast, strong intenders were on average less indulgent compared to weak intenders. The TPB components discriminated strongly between the two groups with partial *eta* coefficients in the range 0.41, *P* < .001 (attitudes), to 0.15 *P* < .001 (Subjective norms). Intention varied systematically with indulgence (eta 0.043, *P* < .05), whereas parents' own oral hygiene behavior did not maintain its significant relationship in the multivariate GLM analyses. Adjusted *R* ^2^ were .061, .015, .047, .157, .393, and .421 for parents' own oral hygiene behavior, knowledge, indulgence, subjective norms, perceived control, and attitudes, respectively.

**Table 6:** Caretakers' attitudes to care for their child's toothbrushing, knowledge, and own hygiene behavior by intention to brush child's teeth

	Strong intention	Weak intention	Partial eta squared
	Mean 95% CI	Mean 95% CI	
Own oral hygiene behavior	8.7(8.2‐9.1)	9.3 (8.7‐9.9)	0.011
Attitudes toward child's toothbrushing	2.7(2.5‐2.8)	4.2(4.0‐4.4)	0.410**
Subjective norm regarding child's toothbrushing	3.1 (2.8‐3.3)	4.3 (4.0‐4.7)	0.154**
Perceived control regarding child's toothbrushing	3.8 (3.6‐4.0)	5.8 (5.5‐6.1)	0.364**
Indulgence regarding child's tooth brushing	8.1 (7.8‐8.4)	7.5 (7.1‐7.8)	0.031*
Knowledge oral health	7.1(6.8‐7.2)	6.9 (6.9‐7.2)	0.001

Note

Multivariate general linear model with intention as a fixed factor. Effect size (partial Eta squared).

Adjusted for sociodemographic characteristics (caretakers age, education, and length of stay in Norway)

Wilk's Lamda: 0.437, *P* < .001.

* *P* < .05.

** *P* < .001.

## 4 DISCUSSION

To our knowledge, this study is the first to examine early toothbrushing intentions and its covariates among non‐Western immigrant parents of children aged 6 months or less. The present findings are consistent with those of previous published studies focusing on immigrant parents of pre‐school children, indicating that unfavorable attitudes and perceptions toward the child's oral health care are associated with parents' lower education, younger ages, and shorter length of residence in Norway.^7^, ^17^ Although immigrants represent a lower socioeconomic status group than native inhabitants in most societies, studies from Scandinavian countries have shown that children's health status varies according to social conditions among participants with and without immigrant status.^4^ Moreover, the present findings showed that the TPB components, attitudes, subjective norms, and perceived behavioral control, were independent significant covariates of parental intention to brush children's teeth, explaining about 41% of its variance. This supports the TPB in predicting immigrant parents' intention to take care of their children's oral health from an early age and is consistent with previous research regarding older pre‐school children's tooth brushing behavior.^20^, ^23^ Moreover, the fit of the TPB model observed in this study accords with that of a TPB meta‐analysis regarding various health‐related behaviors, accounting for 44% of the variance in behavioral intentions.^24^

Socialization theory proposes that the family constitutes the primary socializing agent and is a relevant perspective for understanding how oral health‐related behaviors are adopted among younger children.^25^ From the point of view of Bandura's social cognitive theory,^25^ overt behaviors of parents represent a significant social influence, implying that socialization of children's oral health‐related behaviors is a modeling process. Within the oral health domain, mothers' impact on pre‐school children is recognized to be particularly important.^26^ In accordance with theoretical and empirical evidence, this study revealed that parents with strong intentions to brush their child's teeth performed their own oral hygiene most frequently. This association, however, did not maintain significance in the multivariate analysis suggesting that the influence of parents' own oral hygiene behavior is mediated through more proximal attitudes and perceptions.

As shown in the present results, parents' intention to brush children's teeth was primarily driven by their attitudes, followed in descending order by perceived behavioral control and subjective norms. Thus, parents who perceived favorable consequences accruing from children's regular toothbrushing, who were confident in their ability to circumvent difficulties associated with toothbrushing performance, and who felt a normative pressure were more likely to have strong intentions. The relative importance of attitudes and perceived behavioral control as revealed by this study is consistent with those reported previously when children's oral health behavior has been analyzed in the context of the TPB.^20^, ^23^ In contrast, parents with strong indulgence perceptions were less likely to report toothbrushing intentions. Thus, indulgent parents believed that it is not worth a battle with the child when it comes to regular toothbrushing. A research group in the United Kingdom found that although parents were aware of the importance of regular toothbrushing, the child's behavioral challenges and stress situation of the parents hindered performance of this practice.^27^

A major strength of this study is its theoretical conceptualization and the information provided about behavioral mediators that might constitute valuable tools in the planning of culturally appropriate caries preventive programs. The magnitude of the associations presented and the findings harmonizing with the TPB indicate acceptable reliability and validity of the present findings. Another strength is the demonstration of sociodemographic disparities within the group of immigrant parents. Previous studies have commonly been hindered in conducting stratified analyses by nationality groups due to small number of responders although oral health disparities within the immigrant groups themselves have been expected.^4^ This study also provided a commonly missed opportunity to study the influence of immigrant parents' length of residence in Norway. Nevertheless, the convenience sample utilized is a threat to the external validity and generalizability of the findings as is the inclusion of immigrants from broadly heterogeneous and vastly different ethnic and religious groups. Whereas the measures of sociodemographic factors were developed with the general Norwegian population in mind, they might not accurately reflect the status of immigrants. One main study limitation is the social desirability bias, with over reporting of anticipated preferred and underreporting of anticipated less preferred oral health‐related behaviors. Social desirability is a recognized problem in face‐to‐face interviews.^28^ A tendency toward socially desirable responses might be attributed to the fact that parents were well informed about ways to prevent ECC.

Although parents participating in this study had on average a high knowledge score concerning a child's oral health, their knowledge score did not vary according to sociodemographic factors nor by toothbrushing intentions. Some areas of uncertainty were identified, for instance in terms of ambiguity about when to give children sugared snacks. Moreover, a substantial proportion of parents assured that breastfeeding at night would not lead to dental caries. Similar findings have been reported in other studies.^29^ Cultural and religious traditions encouraging children's breastfeeding up to the age of 2 years should be taken into consideration. Since prolonged breastfeeding was found to be associated with higher odds for dental caries, increased awareness is needed for parental ignorance concerning this practice.^30^

### 4.1 Implications

Early childhood caries might be present shortly after tooth eruption, and caregivers should be educated about children's oral health before and immediately after delivery. This study highlights the importance of tackling social inequalities in attitudes, perceptions, and their own oral hygiene behaviors among immigrant parents, one of the most vulnerable groups in society. To promote children's toothbrushing on a twice a day basis, educational messages should establish and strengthen parents favorable attitudes, target perceptions of the difficulties parents perceive as being associated with this behavior, and their perceived norms. The lack of effect of knowledge on toothbrushing intentions should not be taken as evidence that caregivers' awareness of caries prevention is unimportant. Rather, every opportunity should be taken to reinforce the parent's oral health‐related knowledge and correct any misperceptions identified.

## 5 CONCLUSION

This study was undertaken to investigate oral health knowledge and attitudes among non‐Western immigrant parents visiting health centers located in Bergen, Norway. Overall, non‐Western immigrant parents presented with good oral health‐related knowledge and had favorable attitudes and strong intentions toward children's regular tooth brushing behavior. Parents' knowledge and attitudes varied in the expected direction by social indicators indicating the most vulnerable groups to be prioritized in preventive programs. Moreover, to motivate parents for children's regular tooth brushing, their attitudes, norms, and perceived control seem to be of more importance than their level of factual oral health‐related knowledge. Knowledge of the cultural and habitual contributing factors of ECC can be addressed in prevention policies directed to decrease the burden of this disease and consequently reducing existing oral health inequalities in the Norwegian society.

## CONFLICT OF INTEREST

The authors declare no conflict of interest.

## AUTHOR CONTRIBUTIONS

Manal Mustafa conceived the ideas, methodology, validation, investigation, data collection‐analysis, writing original draft, and project administration. Elwalid F Nasir involved in methodology, validation, analysis, review and editing. Anne Nordrehaug Åstrøm involved in conceptualization, methodology, validation, formal analysis, data curation, and writing—review and editing.

## ETHICAL APPROVAL

Ethical approval was obtained from the Norwegian Local Health District Human Research Ethics Committees (REK) (2015/2037/REK vest). All aspects of the study were in agreement with the latest version of the Helsinki Declaration. Participation was voluntary, based on informed consent, and all participants were allowed to withdraw from the study without giving any reason and without the withdrawal having any negative impact for the individual. Written informed consent was sought according to the strict criteria for health behavior research. The list of names and addresses corresponding to identification numbers was kept separately. In constructing the study interview, efforts have been made to ensure that the questions would not be in conflict with local values and norms. Subjects were invited to participate in the study and those consenting in writing were provided with face‐to‐face administered interview to complete.

## Supporting information

Tab S1

## Data Availability

The datasets generated and analyzed in the study are not publicly available as they contain information that could potentially identify participants and/or health facilities.
