# Supplementary material for: Attitudes toward brushing children's teeth—A study among parents with immigrant status in Norway
Source: Int J Paediatr Dent. 2020 Jul 11;31(1):80–8. doi: 10.1111/ipd.12683 (PMC7818439; doi:10.1111/ipd.12683)
Supplement: Supplementary file 1 — Tab S1 [file IPD-31-80-s001.docx]

Supplementary table

| Country | Number of participants | Percent |
| --- | --- | --- |
| Afghanistan | 12 | 2.6 |
| Algeria | 2 | 0.45 |
| [Azerbaijan](https://history.state.gov/countries/azerbaijan) | 1 | 0.23 |
| [Bangladesh](https://history.state.gov/countries/bangladesh) | 5 | 1.11 |
| [Bosnia and Herzegovina](https://history.state.gov/countries/bosnia-herzegovina) | 4 | 0.87 |
| [Brazil](https://history.state.gov/countries/brazil) | 8 | 1.75 |
| [Dominican Republic](https://history.state.gov/countries/dominican-republic) | 1 | 0.23 |
| [Ecuador](https://history.state.gov/countries/ecuador) | 1 | 0.23 |
| Ivory coast | 1 | 0.23 |
| Eritrea | 28 | 6.11 |
| Ethiopia | 11 | 2.4 |
| Philippines | 18 | 3.83 |
| Gambia | 5 | 1.11 |
| Ghana | 5 | 1.11 |
| [Belarus](https://history.state.gov/countries/russia) | 2 | 0.45 |
| India | 23 | 5.02 |
| Indonesia | 2 | 0.45 |
| [Iraq](https://history.state.gov/countries/iraq) | 18 | 3.83 |
| Iran | 2 | 0.45 |
| China | 15 | 3.28 |
| Republic of the Congo | 4 | 0.87 |
| [Kosovo](https://history.state.gov/countries/kosovo) | 2 | 0.45 |
| Lesotho | 9 | 1.96 |
| Liberia | 5 | 1.11 |
| [Libya](https://history.state.gov/countries/libya) | 1 | 0.23 |
| Lithuania | 34 | 7.42 |
| Morocco | 9 | 1.96 |
| Moldova | 3 | 0.67 |
| [Nepal](https://history.state.gov/countries/nepal) | 2 | 0.45 |
| Nigeria | 1 | 0.23 |
| [Pakistan](https://history.state.gov/countries/nigeria) | 11 | 2.4 |
| Peru | 1 | 0.23 |
| [Romania](https://history.state.gov/countries/peru) | 12 | 2.62 |
| [Russia](https://history.state.gov/countries/russia) | 7 | 1.53 |
| Rwanda | 2 | 0.45 |
| Sierra Leone | 1 | 0.23 |
| Somalia | 39 | 8.52 |
| Sri Lanka | 10 | 2.18 |
| Sudan | 7 | 1.53 |
| Taiwan | 1 | 0.23 |
| Thailand | 8 | 1.75 |
| Ukraine | 1 | 0.23 |
| Vietnam | 12 | 2.62 |
| Poland | 15 | 3.28 |
| Palestine | 3 | 0.67 |
| Norway | 60 | 13.1 |
| Albania | 1 | 0.23 |
| Burma | 3 | 0.67 |
| Yemen | 2 | 0.45 |
| Tanzania | 1 | 0.23 |
| [Syria](https://history.state.gov/countries/syria) | 13 | 2.84 |
| Eastland | 1 | 0.23 |
| Turkey | 2 | 0.45 |
| Germany | 2 | 0.45 |
| Hungary | 1 | 0.23 |
| [Spain](https://history.state.gov/countries/spain) | 2 | 0.45 |
| [Canada](https://history.state.gov/countries/canada) | 2 | 0.45 |
| South Africa | 1 | 0.23 |
| Mexico | 1 | 0.23 |
| Iceland | 1 | 0.23 |
| Chile | 1 | 0.23 |

Number of caretakers (both mothers and fathers) according to the country of origin. Data presented as number of individual and as percent of the total study participants.
